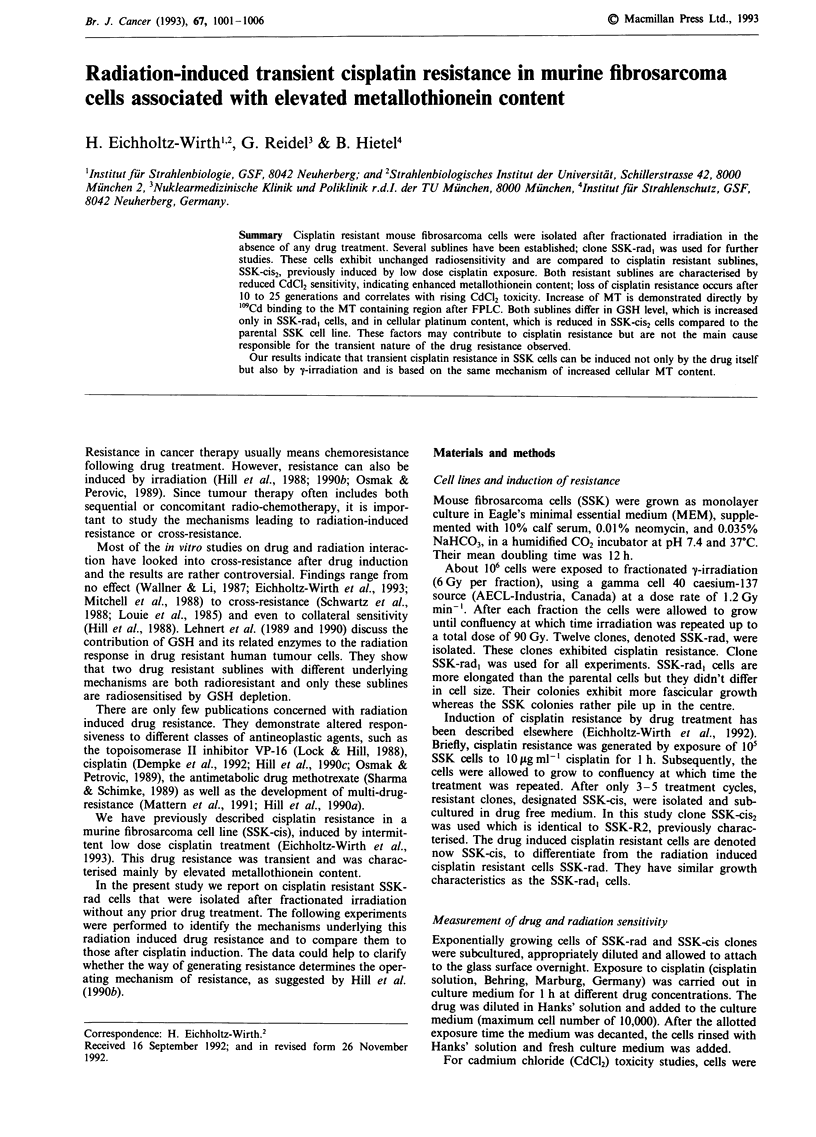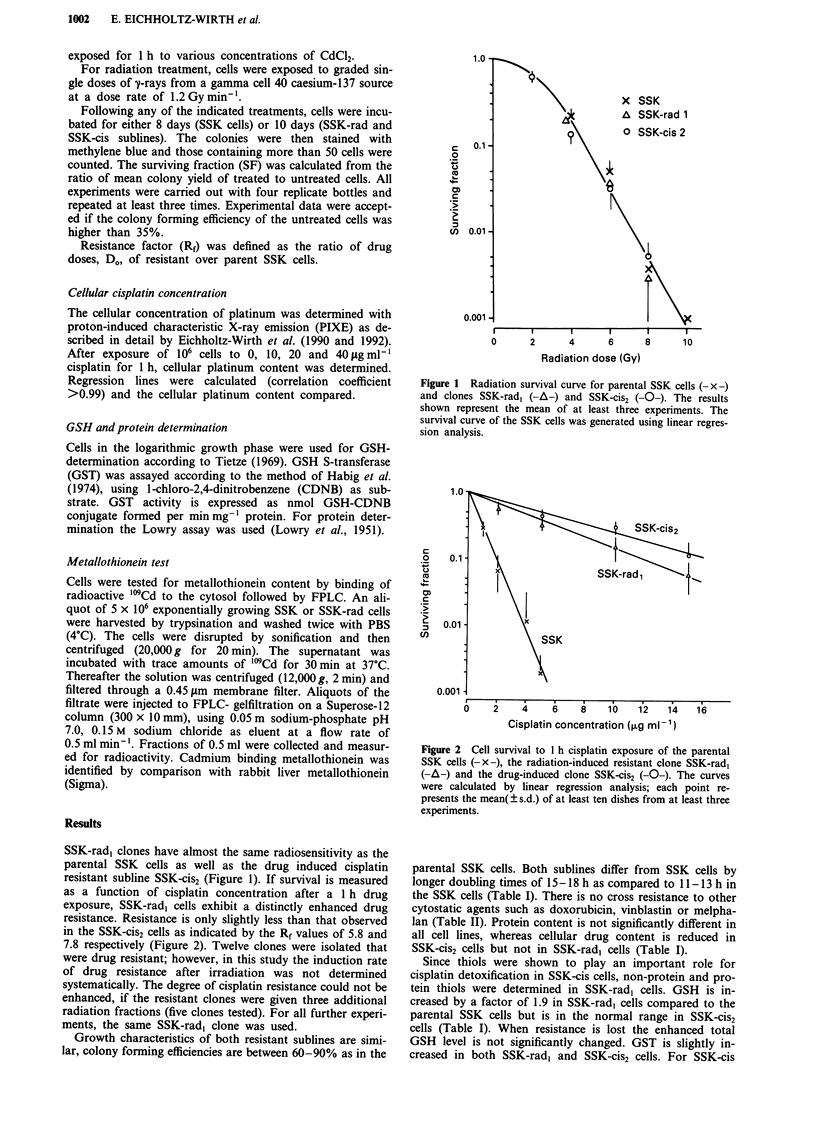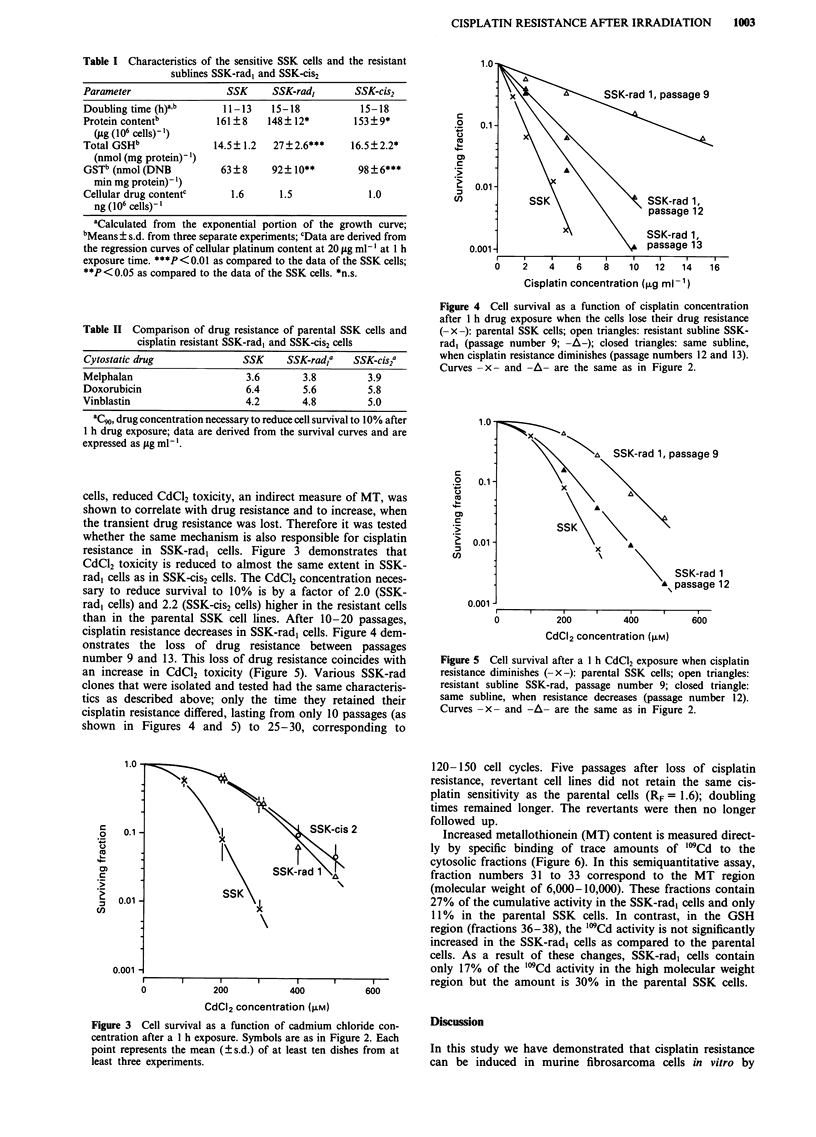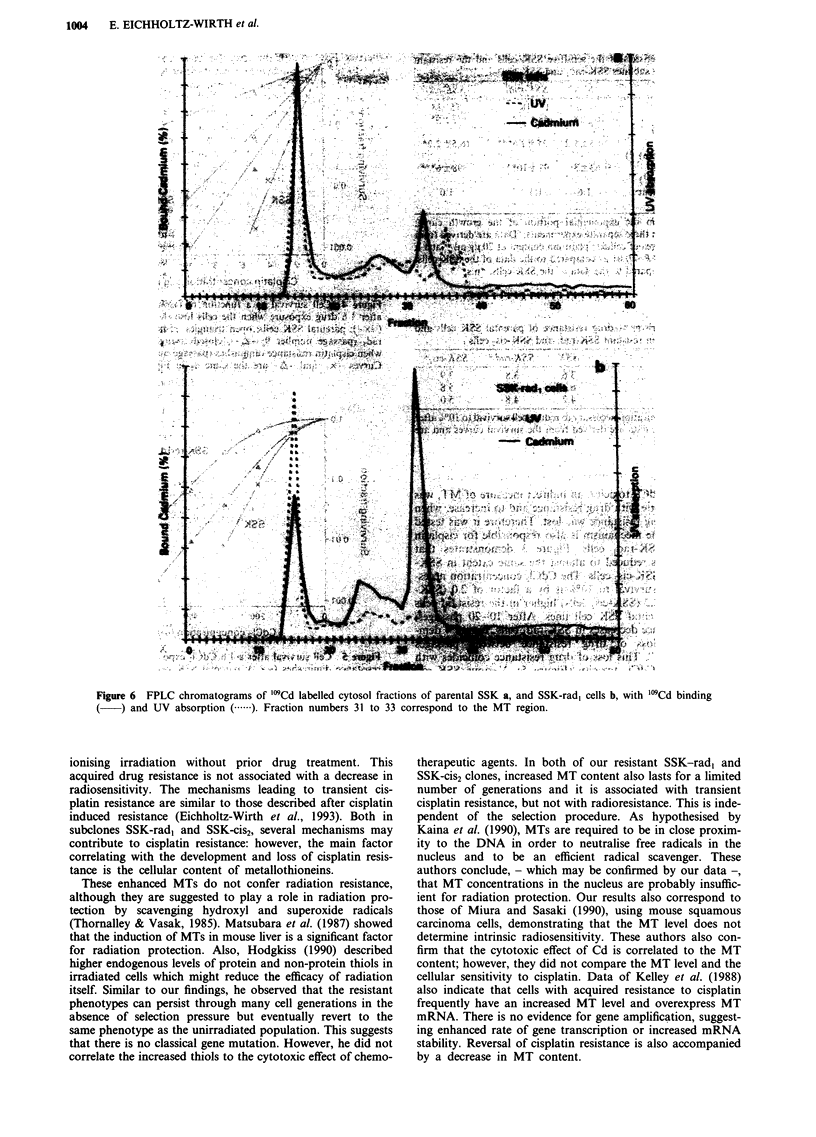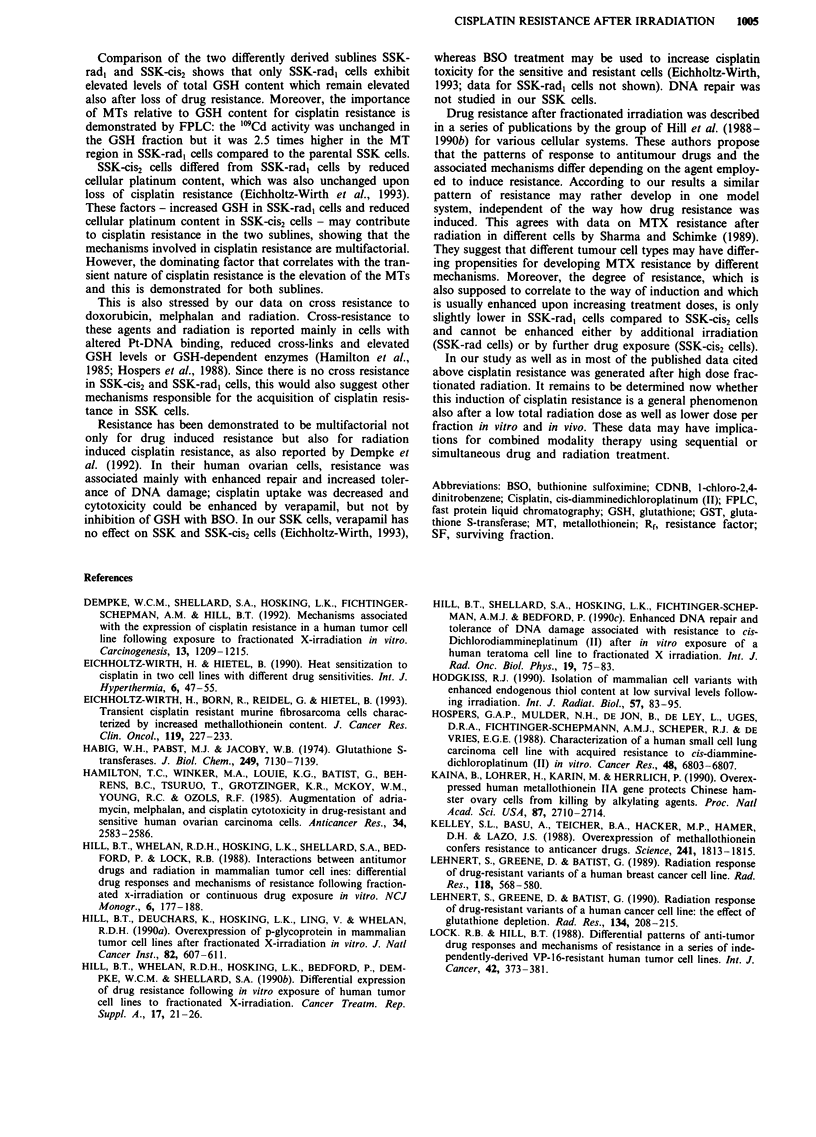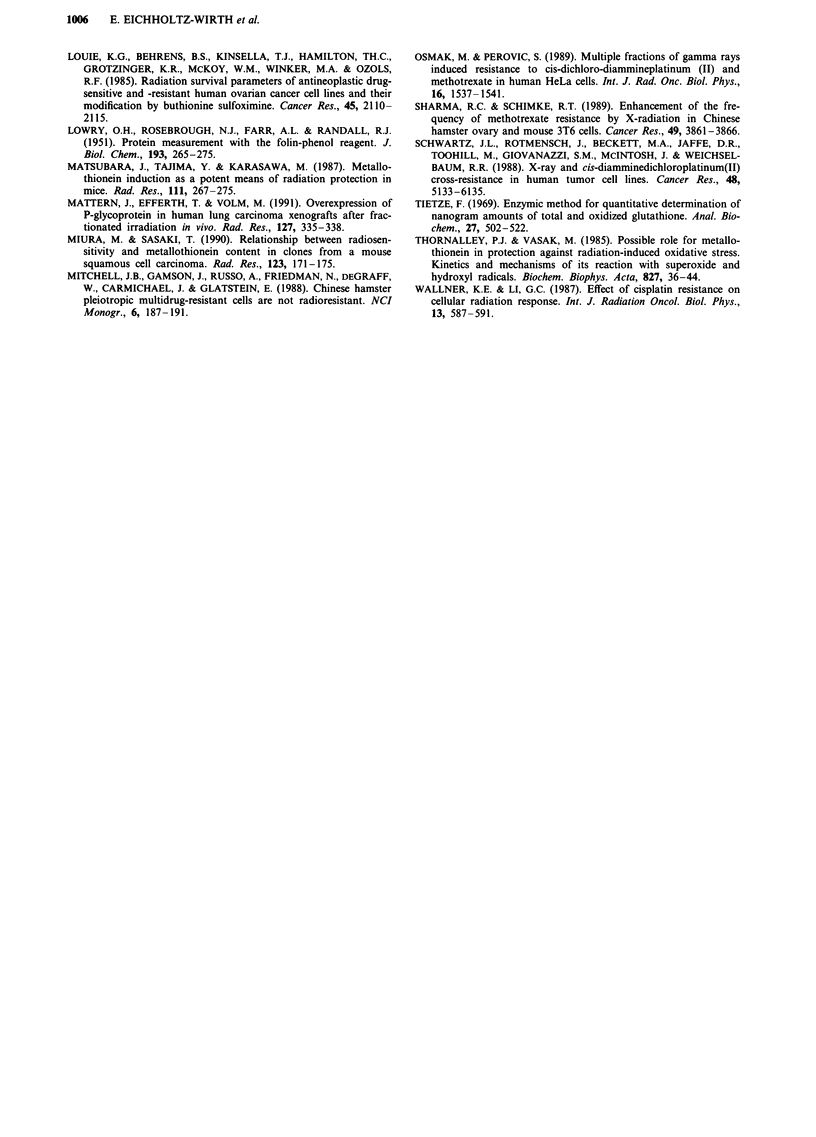# Radiation-induced transient cisplatin resistance in murine fibrosarcoma cells associated with elevated metallothionein content.

**DOI:** 10.1038/bjc.1993.183

**Published:** 1993-05

**Authors:** H. Eichholtz-Wirth, G. Reidel, B. Hietel

**Affiliations:** Institut für Strahlenbiologie, GSF, Neuherberg, Germany.

## Abstract

Cisplatin resistant mouse fibrosarcoma cells were isolated after fractionated irradiation in the absence of any drug treatment. Several sublines have been established; clone SSK-rad1 was used for further studies. These cells exhibit unchanged radiosensitivity and are compared to cisplatin resistant sublines, SSK-cis2, previously induced by low dose cisplatin exposure. Both resistant sublines are characterised by reduced CdCl2 sensitivity, indicating enhanced metallothionein content; loss of cisplatin resistance occurs after 10 to 25 generations and correlates with rising CdCl2 toxicity. Increase of MT is demonstrated directly by 109Cd binding to the MT containing region after FPLC. Both sublines differ in GSH level, which is increased only in SSK-rad1 cells, and in cellular platinum content, which is reduced in SSK-cis2 cells compared to the parental SSK cell line. These factors may contribute to cisplatin resistance but are not the main cause responsible for the transient nature of the drug resistance observed. Our results indicate that transient cisplatin resistance in SSK cells can be induced not only by the drug itself but also by gamma-irradiation and is based on the same mechanism of increased cellular MT content.


Br. J. Cancer (1993), 67, 1001-1006 © Macmillan Press Ltd., 1993

### Radiation-induced transient cisplatin resistance in murine fibrosarcoma cells associated with elevated metallothionein content

H. Eichholtz-Wirth[1,2], G. Reidel[3] & B. Hietel[4]

[1]*Institut für Strahlenbiologie, GSF, 8042 Neuherberg; and* [2]*Strahlenbiologisches Institut der Universität, Schillerstrasse 42, 8000 München 2,* [3]*Nuklearmedizinische Klinik und Poliklinik r.d.I. der TU München, 8000 München,* [4]*Institut für Strahlenschutz, GSF, 8042 Neuherberg, Germany.*

**Summary** Cisplatin resistant mouse fibrosarcoma cells were isolated after fractionated irradiation in the absence of any drug treatment. Several sublines have been established; clone SSK-rad$_1$ was used for further studies. These cells exhibit unchanged radiosensitivity and are compared to cisplatin resistant sublines, SSK-cis$_2$, previously induced by low dose cisplatin exposure. Both resistant sublines are characterised by reduced $CdCl_2$ sensitivity, indicating enhanced metallothionein content; loss of cisplatin resistance occurs after 10 to 25 generations and correlates with rising $CdCl_2$ toxicity. Increase of MT is demonstrated directly by $^{109}$Cd binding to the MT containing region after FPLC. Both sublines differ in GSH level, which is increased only in SSK-rad$_1$ cells, and in cellular platinum content, which is reduced in SSK-cis$_2$ cells compared to the parental SSK cell line. These factors may contribute to cisplatin resistance but are not the main cause responsible for the transient nature of the drug resistance observed.

Our results indicate that transient cisplatin resistance in SSK cells can be induced not only by the drug itself but also by γ-irradiation and is based on the same mechanism of increased cellular MT content.

Resistance in cancer therapy usually means chemoresistance following drug treatment. However, resistance can also be induced by irradiation (Hill *et al.*, 1988; 1990*b*; Osmak & Perovic, 1989). Since tumour therapy often includes both sequential or concomitant radio-chemotherapy, it is important to study the mechanisms leading to radiation-induced resistance or cross-resistance.

Most of the *in vitro* studies on drug and radiation interaction have looked into cross-resistance after drug induction and the results are rather controversial. Findings range from no effect (Wallner & Li, 1987; Eichholtz-Wirth *et al.*, 1993; Mitchell *et al.*, 1988) to cross-resistance (Schwartz *et al.*, 1988; Louie *et al.*, 1985) and even to collateral sensitivity (Hill *et al.*, 1988). Lehnert *et al.* (1989 and 1990) discuss the contribution of GSH and its related enzymes to the radiation response in drug resistant human tumour cells. They show that two drug resistant sublines with different underlying mechanisms are both radioresistant and only these sublines are radiosensitised by GSH depletion.

There are only few publications concerned with radiation induced drug resistance. They demonstrate altered responsiveness to different classes of antineoplastic agents, such as the topoisomerase II inhibitor VP-16 (Lock & Hill, 1988), cisplatin (Dempke *et al.*, 1992; Hill *et al.*, 1990*c*; Osmak & Petrovic, 1989), the antimetabolic drug methotrexate (Sharma & Schimke, 1989) as well as the development of multi-drug-resistance (Mattern *et al.*, 1991; Hill *et al.*, 1990*a*).

We have previously described cisplatin resistance in a murine fibrosarcoma cell line (SSK-cis), induced by intermittent low dose cisplatin treatment (Eichholtz-Wirth *et al.*, 1993). This drug resistance was transient and was characterised mainly by elevated metallothionein content.

In the present study we report on cisplatin resistant SSK-rad cells that were isolated after fractionated irradiation without any prior drug treatment. The following experiments were performed to identify the mechanisms underlying this radiation induced drug resistance and to compare them to those after cisplatin induction. The data could help to clarify whether the way of generating resistance determines the operating mechanism of resistance, as suggested by Hill *et al.* (1990*b*).

Correspondence: H. Eichholtz-Wirth.[2]
Received 16 September 1992; and in revised form 26 November 1992.

### Materials and methods

### *Cell lines and induction of resistance*

Mouse fibrosarcoma cells (SSK) were grown as monolayer culture in Eagle's minimal essential medium (MEM), supplemented with 10% calf serum, 0.01% neomycin, and 0.035% $NaHCO_3$, in a humidified $CO_2$ incubator at pH 7.4 and 37°C. Their mean doubling time was 12 h.

About $10^6$ cells were exposed to fractionated γ-irradiation (6 Gy per fraction), using a gamma cell 40 caesium-137 source (AECL-Industria, Canada) at a dose rate of 1.2 Gy min$^{-1}$. After each fraction the cells were allowed to grow until confluency at which time irradiation was repeated up to a total dose of 90 Gy. Twelve clones, denoted SSK-rad, were isolated. These clones exhibited cisplatin resistance. Clone SSK-rad$_1$ was used for all experiments. SSK-rad$_1$ cells are more elongated than the parental cells but they didn't differ in cell size. Their colonies exhibit more fascicular growth whereas the SSK colonies rather pile up in the centre.

Induction of cisplatin resistance by drug treatment has been described elsewhere (Eichholtz-Wirth *et al.*, 1992). Briefly, cisplatin resistance was generated by exposure of $10^5$ SSK cells to 10 μg ml$^{-1}$ cisplatin for 1 h. Subsequently, the cells were allowed to grow to confluency at which time the treatment was repeated. After only 3-5 treatment cycles, resistant clones, designated SSK-cis, were isolated and subcultured in drug free medium. In this study clone SSK-cis$_2$ was used which is identical to SSK-R2, previously characterised. The drug induced cisplatin resistant cells are denoted now SSK-cis, to differentiate from the radiation induced cisplatin resistant cells SSK-rad. They have similar growth characteristics as the SSK-rad$_1$ cells.

### *Measurement of drug and radiation sensitivity*

Exponentially growing cells of SSK-rad and SSK-cis clones were subcultured, appropriately diluted and allowed to attach to the glass surface overnight. Exposure to cisplatin (cisplatin solution, Behring, Marburg, Germany) was carried out in culture medium for 1 h at different drug concentrations. The drug was diluted in Hanks' solution and added to the culture medium (maximum cell number of 10,000). After the allotted exposure time the medium was decanted, the cells rinsed with Hanks' solution and fresh culture medium was added.

For cadmium chloride ($CdCl_2$) toxicity studies, cells were exposed for 1 h to various concentrations of $CdCl_2$.

For radiation treatment, cells were exposed to graded single doses of γ-rays from a gamma cell 40 caesium-137 source at a dose rate of $1.2\ Gy\ min^{-1}$.

Following any of the indicated treatments, cells were incubated for either 8 days (SSK cells) or 10 days (SSK-rad and SSK-cis sublines). The colonies were then stained with methylene blue and those containing more than 50 cells were counted. The surviving fraction (SF) was calculated from the ratio of mean colony yield of treated to untreated cells. All experiments were carried out with four replicate bottles and repeated at least three times. Experimental data were accepted if the colony forming efficiency of the untreated cells was higher than 35%.

Resistance factor ($R_f$) was defined as the ratio of drug doses, $D_o$, of resistant over parent SSK cells.

### *Cellular cisplatin concentration*

The cellular concentration of platinum was determined with proton-induced characteristic X-ray emission (PIXE) as described in detail by Eichholtz-Wirth *et al.* (1990 and 1992). After exposure of $10^6$ cells to 0, 10, 20 and $40\ \mu g\ ml^{-1}$ cisplatin for 1 h, cellular platinum content was determined. Regression lines were calculated (correlation coefficient >0.99) and the cellular platinum content compared.

### *GSH and protein determination*

Cells in the logarithmic growth phase were used for GSH-determination according to Tietze (1969). GSH S-transferase (GST) was assayed according to the method of Habig *et al.* (1974), using 1-chloro-2,4-dinitrobenzene (CDNB) as substrate. GST activity is expressed as nmol GSH-CDNB conjugate formed per min $mg^{-1}$ protein. For protein determination the Lowry assay was used (Lowry *et al.*, 1951).

### *Metallothionein test*

Cells were tested for metallothionein content by binding of radioactive $^{109}Cd$ to the cytosol followed by FPLC. An aliquot of $5 \times 10^6$ exponentially growing SSK or SSK-rad cells were harvested by trypsination and washed twice with PBS (4°C). The cells were disrupted by sonification and then centrifuged (20,000 *g* for 20 min). The supernatant was incubated with trace amounts of $^{109}Cd$ for 30 min at 37°C. Thereafter the solution was centrifuged (12,000 *g*, 2 min) and filtered through a 0.45 μm membrane filter. Aliquots of the filtrate were injected to FPLC- gelfiltration on a Superose-12 column (300 × 10 mm), using 0.05 m sodium-phosphate pH 7.0, 0.15 M sodium chloride as eluent at a flow rate of $0.5\ ml\ min^{-1}$. Fractions of 0.5 ml were collected and measured for radioactivity. Cadmium binding metallothionein was identified by comparison with rabbit liver metallothionein (Sigma).

**Figure 1** Radiation survival curve for parental SSK cells (–×–) and clones SSK-$rad_1$ (–Δ–) and SSK-$cis_2$ (–O–). The results shown represent the mean of at least three experiments. The survival curve of the SSK cells was generated using linear regression analysis.

**Figure 2** Cell survival to 1 h cisplatin exposure of the parental SSK cells (–×–), the radiation-induced resistant clone SSK-$rad_1$ (–Δ–) and the drug-induced clone SSK-$cis_2$ (–O–). The curves were calculated by linear regression analysis; each point represents the mean(±s.d.) of at least ten dishes from at least three experiments.

### Results

SSK-$rad_1$ clones have almost the same radiosensitivity as the parental SSK cells as well as the drug induced cisplatin resistant subline SSK-$cis_2$ (Figure 1). If survival is measured as a function of cisplatin concentration after a 1 h drug exposure, SSK-$rad_1$ cells exhibit a distinctly enhanced drug resistance. Resistance is only slightly less than that observed in the SSK-$cis_2$ cells as indicated by the $R_f$ values of 5.8 and 7.8 respectively (Figure 2). Twelve clones were isolated that were drug resistant; however, in this study the induction rate of drug resistance after irradiation was not determined systematically. The degree of cisplatin resistance could not be enhanced, if the resistant clones were given three additional radiation fractions (five clones tested). For all further experiments, the same SSK-$rad_1$ clone was used.

Growth characteristics of both resistant sublines are similar, colony forming efficiencies are between 60–90% as in the parental SSK cells. Both sublines differ from SSK cells by longer doubling times of 15–18 h as compared to 11–13 h in the SSK cells (Table I). There is no cross resistance to other cytostatic agents such as doxorubicin, vinblastin or melphalan (Table II). Protein content is not significantly different in all cell lines, whereas cellular drug content is reduced in SSK-$cis_2$ cells but not in SSK-$rad_1$ cells (Table I).

Since thiols were shown to play an important role for cisplatin detoxification in SSK-cis cells, non-protein and protein thiols were determined in SSK-$rad_1$ cells. GSH is increased by a factor of 1.9 in SSK-$rad_1$ cells compared to the parental SSK cells but is in the normal range in SSK-$cis_2$ cells (Table I). When resistance is lost the enhanced total GSH level is not significantly changed. GST is slightly increased in both SSK-$rad_1$ and SSK-$cis_2$ cells. For SSK-cis

**Table I** Characteristics of the sensitive SSK cells and the resistant sublines SSK-rad$_1$ and SSK-cis$_2$

| *Parameter* | *SSK* | *SSK-rad$_1$* | *SSK-cis$_2$* |
|---|---|---|---|
| Doubling time (h)[a,b] | 11–13 | 15–18 | 15–18 |
| Protein content[b] ($\mu$g (10$^6$ cells)$^{-1}$) | 161±8 | 148±12* | 153±9* |
| Total GSH[b] (nmol (mg protein)$^{-1}$) | 14.5±1.2 | 27±2.6*** | 16.5±2.2* |
| GST[b] (nmol (DNB min mg protein)$^{-1}$) | 63±8 | 92±10** | 98±6*** |
| Cellular drug content[c] ng (10$^6$ cells)$^{-1}$ | 1.6 | 1.5 | 1.0 |

[a]Calculated from the exponential portion of the growth curve; [b]Means±s.d. from three separate experiments; [c]Data are derived from the regression curves of cellular platinum content at 20 $\mu$g ml$^{-1}$ at 1 h exposure time. ***$P<0.01$ as compared to the data of the SSK cells; **$P<0.05$ as compared to the data of the SSK cells. *n.s.

**Table II** Comparison of drug resistance of parental SSK cells and cisplatin resistant SSK-rad$_1$ and SSK-cis$_2$ cells

| *Cytostatic drug* | *SSK* | *SSK-rad$_1$[a]* | *SSK-cis$_2$[a]* |
|---|---|---|---|
| Melphalan | 3.6 | 3.8 | 3.9 |
| Doxorubicin | 6.4 | 5.6 | 5.8 |
| Vinblastin | 4.2 | 4.8 | 5.0 |

[a]$C_{90}$, drug concentration necessary to reduce cell survival to 10% after 1 h drug exposure; data are derived from the survival curves and are expressed as $\mu$g ml$^{-1}$.

cells, reduced $CdCl_2$ toxicity, an indirect measure of MT, was shown to correlate with drug resistance and to increase, when the transient drug resistance was lost. Therefore it was tested whether the same mechanism is also responsible for cisplatin resistance in SSK-rad$_1$ cells. Figure 3 demonstrates that $CdCl_2$ toxicity is reduced to almost the same extent in SSK-rad$_1$ cells as in SSK-cis$_2$ cells. The $CdCl_2$ concentration necessary to reduce survival to 10% is by a factor of 2.0 (SSK-rad$_1$ cells) and 2.2 (SSK-cis$_2$ cells) higher in the resistant cells than in the parental SSK cell lines. After 10–20 passages, cisplatin resistance decreases in SSK-rad$_1$ cells. Figure 4 demonstrates the loss of drug resistance between passages number 9 and 13. This loss of drug resistance coincides with an increase in $CdCl_2$ toxicity (Figure 5). Various SSK-rad clones that were isolated and tested had the same characteristics as described above; only the time they retained their cisplatin resistance differed, lasting from only 10 passages (as shown in Figures 4 and 5) to 25–30, corresponding to

**Figure 3** Cell survival as a function of cadmium chloride concentration after a 1 h exposure. Symbols are as in Figure 2. Each point represents the mean (±s.d.) of at least ten dishes from at least three experiments.

**Figure 4** Cell survival as a function of cisplatin concentration after 1 h drug exposure when the cells lose their drug resistance (–×–): parental SSK cells; open triangles: resistant subline SSK-rad$_1$ (passage number 9; –Δ–); closed triangles: same subline, when cisplatin resistance diminishes (passage numbers 12 and 13). Curves –×– and –Δ– are the same as in Figure 2.

**Figure 5** Cell survival after a 1 h $CdCl_2$ exposure when cisplatin resistance diminishes (–×–): parental SSK cells; open triangles: resistant subline SSK-rad, passage number 9; closed triangle: same subline, when resistance decreases (passage number 12). Curves –×– and –Δ– are the same as in Figure 2.

120–150 cell cycles. Five passages after loss of cisplatin resistance, revertant cell lines did not retain the same cisplatin sensitivity as the parental cells ($R_F = 1.6$); doubling times remained longer. The revertants were then no longer followed up.

Increased metallothionein (MT) content is measured directly by specific binding of trace amounts of $^{109}Cd$ to the cytosolic fractions (Figure 6). In this semiquantitative assay, fraction numbers 31 to 33 correspond to the MT region (molecular weight of 6,000–10,000). These fractions contain 27% of the cumulative activity in the SSK-rad$_1$ cells and only 11% in the parental SSK cells. In contrast, in the GSH region (fractions 36–38), the $^{109}Cd$ activity is not significantly increased in the SSK-rad$_1$ cells as compared to the parental cells. As a result of these changes, SSK-rad$_1$ cells contain only 17% of the $^{109}Cd$ activity in the high molecular weight region but the amount is 30% in the parental SSK cells.

### Discussion

In this study we have demonstrated that cisplatin resistance can be induced in murine fibrosarcoma cells *in vitro* by

**Figure 6** FPLC chromatograms of $^{109}Cd$ labelled cytosol fractions of parental SSK **a**, and SSK-rad$_1$ cells **b**, with $^{109}Cd$ binding (——) and UV absorption (······). Fraction numbers 31 to 33 correspond to the MT region.

ionising irradiation without prior drug treatment. This acquired drug resistance is not associated with a decrease in radiosensitivity. The mechanisms leading to transient cisplatin resistance are similar to those described after cisplatin induced resistance (Eichholtz-Wirth *et al.*, 1993). Both in subclones SSK-rad$_1$ and SSK-cis$_2$, several mechanisms may contribute to cisplatin resistance: however, the main factor correlating with the development and loss of cisplatin resistance is the cellular content of metallothioneins.

These enhanced MTs do not confer radiation resistance, although they are suggested to play a role in radiation protection by scavenging hydroxyl and superoxide radicals (Thornalley & Vasak, 1985). Matsubara *et al.* (1987) showed that the induction of MTs in mouse liver is a significant factor for radiation protection. Also, Hodgkiss (1990) described higher endogenous levels of protein and non-protein thiols in irradiated cells which might reduce the efficacy of radiation itself. Similar to our findings, he observed that the resistant phenotypes can persist through many cell generations in the absence of selection pressure but eventually revert to the same phenotype as the unirradiated population. This suggests that there is no classical gene mutation. However, he did not correlate the increased thiols to the cytotoxic effect of chemotherapeutic agents. In both of our resistant SSK–rad$_1$ and SSK-cis$_2$ clones, increased MT content also lasts for a limited number of generations and it is associated with transient cisplatin resistance, but not with radioresistance. This is independent of the selection procedure. As hypothesised by Kaina *et al.* (1990), MTs are required to be in close proximity to the DNA in order to neutralise free radicals in the nucleus and to be an efficient radical scavenger. These authors conclude, – which may be confirmed by our data –, that MT concentrations in the nucleus are probably insufficient for radiation protection. Our results also correspond to those of Miura and Sasaki (1990), using mouse squamous carcinoma cells, demonstrating that the MT level does not determine intrinsic radiosensitivity. These authors also confirm that the cytotoxic effect of Cd is correlated to the MT content; however, they did not compare the MT level and the cellular sensitivity to cisplatin. Data of Kelley *et al.* (1988) also indicate that cells with acquired resistance to cisplatin frequently have an increased MT level and overexpress MT mRNA. There is no evidence for gene amplification, suggesting enhanced rate of gene transcription or increased mRNA stability. Reversal of cisplatin resistance is also accompanied by a decrease in MT content.

Comparison of the two differently derived sublines SSK-$rad_1$ and SSK-$cis_2$ shows that only SSK-$rad_1$ cells exhibit elevated levels of total GSH content which remain elevated also after loss of drug resistance. Moreover, the importance of MTs relative to GSH content for cisplatin resistance is demonstrated by FPLC: the $^{109}Cd$ activity was unchanged in the GSH fraction but it was 2.5 times higher in the MT region in SSK-$rad_1$ cells compared to the parental SSK cells.

SSK-$cis_2$ cells differed from SSK-$rad_1$ cells by reduced cellular platinum content, which was also unchanged upon loss of cisplatin resistance (Eichholtz-Wirth *et al.*, 1993). These factors – increased GSH in SSK-$rad_1$ cells and reduced cellular platinum content in SSK-$cis_2$ cells – may contribute to cisplatin resistance in the two sublines, showing that the mechanisms involved in cisplatin resistance are multifactorial. However, the dominating factor that correlates with the transient nature of cisplatin resistance is the elevation of the MTs and this is demonstrated for both sublines.

This is also stressed by our data on cross resistance to doxorubicin, melphalan and radiation. Cross-resistance to these agents and radiation is reported mainly in cells with altered Pt-DNA binding, reduced cross-links and elevated GSH levels or GSH-dependent enzymes (Hamilton *et al.*, 1985; Hospers *et al.*, 1988). Since there is no cross resistance in SSK-$cis_2$ and SSK-$rad_1$ cells, this would also suggest other mechanisms responsible for the acquisition of cisplatin resistance in SSK cells.

Resistance has been demonstrated to be multifactorial not only for drug induced resistance but also for radiation induced cisplatin resistance, as also reported by Dempke *et al.* (1992). In their human ovarian cells, resistance was associated mainly with enhanced repair and increased tolerance of DNA damage; cisplatin uptake was decreased and cytotoxicity could be enhanced by verapamil, but not by inhibition of GSH with BSO. In our SSK cells, verapamil has no effect on SSK and SSK-$cis_2$ cells (Eichholtz-Wirth, 1993), whereas BSO treatment may be used to increase cisplatin toxicity for the sensitive and resistant cells (Eichholtz-Wirth, 1993; data for SSK-$rad_1$ cells not shown). DNA repair was not studied in our SSK cells.

Drug resistance after fractionated irradiation was described in a series of publications by the group of Hill *et al.* (1988–1990*b*) for various cellular systems. These authors propose that the patterns of response to antitumour drugs and the associated mechanisms differ depending on the agent employed to induce resistance. According to our results a similar pattern of resistance may rather develop in one model system, independent of the way how drug resistance was induced. This agrees with data on MTX resistance after radiation in different cells by Sharma and Schimke (1989). They suggest that different tumour cell types may have differing propensities for developing MTX resistance by different mechanisms. Moreover, the degree of resistance, which is also supposed to correlate to the way of induction and which is usually enhanced upon increasing treatment doses, is only slightly lower in SSK-$rad_1$ cells compared to SSK-$cis_2$ cells and cannot be enhanced either by additional irradiation (SSK-rad cells) or by further drug exposure (SSK-$cis_2$ cells).

In our study as well as in most of the published data cited above cisplatin resistance was generated after high dose fractionated radiation. It remains to be determined now whether this induction of cisplatin resistance is a general phenomenon also after a low total radiation dose as well as lower dose per fraction *in vitro* and *in vivo*. These data may have implications for combined modality therapy using sequential or simultaneous drug and radiation treatment.

Abbreviations: BSO, buthionine sulfoximine; CDNB, 1-chloro-2,4-dinitrobenzene; Cisplatin, cis-diamminedichloroplatinum (II); FPLC, fast protein liquid chromatography; GSH, glutathione; GST, glutathione S-transferase; MT, metallothionein; $R_f$, resistance factor; SF, surviving fraction.

### References

DEMPKE, W.C.M., SHELLARD, S.A., HOSKING, L.K., FICHTINGER-SCHEPMAN, A.M. & HILL, B.T. (1992). Mechanisms associated with the expression of cisplatin resistance in a human tumor cell line following exposure to fractionated X-irradiation *in vitro*. *Carcinogenesis*, **13**, 1209–1215.

EICHHOLTZ-WIRTH, H. & HIETEL, B. (1990). Heat sensitization to cisplatin in two cell lines with different drug sensitivities. *Int. J. Hyperthermia*, **6**, 47–55.

EICHHOLTZ-WIRTH, H., BORN, R., REIDEL, G. & HIETEL, B. (1993). Transient cisplatin resistant murine fibrosarcoma cells characterized by increased methallothionein content. *J. Cancer Res. Clin. Oncol.*, **119**, 227–233.

HABIG, W.H., PABST, M.J. & JACOBY, W.B. (1974). Glutathione S-transferases. *J. Biol. Chem.*, **249**, 7130–7139.

HAMILTON, T.C., WINKER, M.A., LOUIE, K.G., BATIST, G., BEHRENS, B.C., TSURUO, T., GROTZINGER, K.R., MCKOY, W.M., YOUNG, R.C. & OZOLS, R.F. (1985). Augmentation of adriamycin, melphalan, and cisplatin cytotoxicity in drug-resistant and sensitive human ovarian carcinoma cells. *Anticancer Res.*, **34**, 2583–2586.

HILL, B.T., WHELAN, R.D.H., HOSKING, L.K., SHELLARD, S.A., BEDFORD, P. & LOCK, R.B. (1988). Interactions between antitumor drugs and radiation in mammalian tumor cell ines: differential drug responses and mechanisms of resistance following fractionated x-irradiation or continuous drug exposure *in vitro*. *NCJ Monogr.*, **6**, 177–188.

HILL, B.T., DEUCHARS, K., HOSKING, L.K., LING, V. & WHELAN, R.D.H. (1990*a*). Overexpression of p-glycoprotein in mammalian tumor cell lines after fractionated X-irradiation *in vitro*. *J. Natl Cancer Inst.*, **82**, 607–611.

HILL, B.T., WHELAN, R.D.H., HOSKING, L.K., BEDFORD, P., DEMPKE, W.C.M. & SHELLARD, S.A. (1990*b*). Differential expression of drug resistance following *in vitro* exposure of human tumor cell lines to fractionated X-irradiation. *Cancer Treatm. Rep. Suppl. A.*, **17**, 21–26.

HILL, B.T., SHELLARD, S.A., HOSKING, L.K., FICHTINGER-SCHEPMAN, A.M.J. & BEDFORD, P. (1990*c*). Enhanced DNA repair and tolerance of DNA damage associated with resistance to *cis*-Dichlorodiammineplatinum (II) after *in vitro* exposure of a human teratoma cell line to fractionated X irradiation. *Int. J. Rad. Onc. Biol. Phys.*, **19**, 75–83.

HODGKISS, R.J. (1990). Isolation of mammalian cell variants with enhanced endogenous thiol content at low survival levels following irradiation. *Int. J. Radiat. Biol.*, **57**, 83–95.

HOSPERS, G.A.P., MULDER, N.H., DE JON, B., DE LEY, L., UGES, D.R.A., FICHTINGER-SCHEPMANN, A.M.J., SCHEPER, R.J. & DE VRIES, E.G.E. (1988). Characterization of a human small cell lung carcinoma cell line with acquired resistance to *cis*-diamminedichloroplatinum (II) *in vitro*. *Cancer Res.*, **48**, 6803–6807.

KAINA, B., LOHRER, H., KARIN, M. & HERRLICH, P. (1990). Overexpressed human metallothionein IIA gene protects Chinese hamster ovary cells from killing by alkylating agents. *Proc. Natl Acad. Sci. USA*, **87**, 2710–2714.

KELLEY, S.L., BASU, A., TEICHER, B.A., HACKER, M.P., HAMER, D.H. & LAZO, J.S. (1988). Overexpression of methallothionein confers resistance to anticancer drugs. *Science*, **241**, 1813–1815.

LEHNERT, S., GREENE, D. & BATIST, G. (1989). Radiation response of drug-resistant variants of a human breast cancer cell line. *Rad. Res.*, **118**, 568–580.

LEHNERT, S., GREENE, D. & BATIST, G. (1990). Radiation response of drug-resistant variants of a human cancer cell line: the effect of glutathione depletion. *Rad. Res.*, **134**, 208–215.

LOCK. R.B. & HILL, B.T. (1988). Differential patterns of anti-tumor drug responses and mechanisms of resistance in a series of independently-derived VP-16-resistant human tumor cell lines. *Int. J. Cancer*, **42**, 373–381.

LOUIE, K.G., BEHRENS, B.S., KINSELLA, T.J., HAMILTON, TH.C., GROTZINGER, K.R., MCKOY, W.M., WINKER, M.A. & OZOLS, R.F. (1985). Radiation survival parameters of antineoplastic drug-sensitive and -resistant human ovarian cancer cell lines and their modification by buthionine sulfoximine. *Cancer Res.*, **45**, 2110–2115.

LOWRY, O.H., ROSEBROUGH, N.J., FARR, A.L. & RANDALL, R.J. (1951). Protein measurement with the folin-phenol reagent. *J. Biol. Chem.*, **193**, 265–275.

MATSUBARA, J., TAJIMA, Y. & KARASAWA, M. (1987). Metallothionein induction as a potent means of radiation protection in mice. *Rad. Res.*, **111**, 267–275.

MATTERN, J., EFFERTH, T. & VOLM, M. (1991). Overexpression of P-glycoprotein in human lung carcinoma xenografts after fractionated irradiation *in vivo*. *Rad. Res.*, **127**, 335–338.

MIURA, M. & SASAKI, T. (1990). Relationship between radiosensitivity and metallothionein content in clones from a mouse squamous cell carcinoma. *Rad. Res.*, **123**, 171–175.

MITCHELL, J.B., GAMSON, J., RUSSO, A., FRIEDMAN, N., DEGRAFF, W., CARMICHAEL, J. & GLATSTEIN, E. (1988). Chinese hamster pleiotropic multidrug-resistant cells are not radioresistant. *NCI Monogr.*, **6**, 187–191.

OSMAK, M. & PEROVIC, S. (1989). Multiple fractions of gamma rays induced resistance to cis-dichloro-diammineplatinum (II) and methotrexate in human HeLa cells. *Int. J. Rad. Onc. Biol. Phys.*, **16**, 1537–1541.

SHARMA, R.C. & SCHIMKE, R.T. (1989). Enhancement of the frequency of methotrexate resistance by X-radiation in Chinese hamster ovary and mouse 3T6 cells. *Cancer Res.*, **49**, 3861–3866.

SCHWARTZ, J.L., ROTMENSCH, J., BECKETT, M.A., JAFFE, D.R., TOOHILL, M., GIOVANAZZI, S.M., MCINTOSH, J. & WEICHSELBAUM, R.R. (1988). X-ray and *cis*-diamminedichloroplatinum(II) cross-resistance in human tumor cell lines. *Cancer Res.*, **48**, 5133–6135.

TIETZE, F. (1969). Enzymic method for quantitative determination of nanogram amounts of total and oxidized glutathione. *Anal. Biochem.*, **27**, 502–522.

THORNALLEY, P.J. & VASAK, M. (1985). Possible role for metallothionein in protection against radiation-induced oxidative stress. Kinetics and mechanisms of its reaction with superoxide and hydroxyl radicals. *Biochem. Biophys. Acta*, **827**, 36–44.

WALLNER, K.E. & LI, G.C. (1987). Effect of cisplatin resistance on cellular radiation response. *Int. J. Radiation Oncol. Biol. Phys.*, **13**, 587–591.